# Ceramic transition metal diboride superlattices with improved ductility and fracture toughness screened by ab initio calculations

**DOI:** 10.1038/s41598-023-39997-4

**Published:** 2023-08-08

**Authors:** Tomáš Fiantok, Nikola Koutná, Davide G. Sangiovanni, Marián Mikula

**Affiliations:** 1 Detached Workplace of Faculty of Mathematics, Physics and Informatics, Comenius University in Bratislava, Turany, Slovakia; 2 Department of Experimental Physics, Faculty of Mathematics, Physics and Informatics, Comenius University in Bratislava, Bratislava, Slovakia; 3 Department of Physics, Chemistry, and Biology (IFM), Linköping University, Linköping, Sweden; 4 Institute of Materials Science and Technology, TU Wien, Vienna, Austria; 5 Institute of Materials and Machine Mechanics SAS, Bratislava, Slovakia

**Keywords:** Condensed-matter physics, Theory and computation, Ceramics, Mechanical properties

## Abstract

Inherent brittleness, which easily leads to crack formation and propagation during use, is a serious problem for protective ceramic thin-film applications. Superlattice architectures, with alternating nm-thick layers of typically softer/stiffer materials, have been proven powerful method to improve the mechanical performance of, e.g., cubic transition metal nitride ceramics. Using high-throughput first-principles calculations, we propose that superlattice structures hold promise also for enhancing mechanical properties and fracture resistance of transition metal diborides with two competing hexagonal phases, $\alpha$ and $\omega$. We study 264 possible combinations of $\alpha /\alpha$, $\alpha /\omega$ or $\omega /\omega$ MB$_2$ (where M $=$ Al or group 3–6 transition metal) diboride superlattices. Based on energetic stability considerations, together with restrictions for lattice and shear modulus mismatch ($\Delta a<4\%$, $\Delta G>40$ GPa), we select 33 superlattice systems for further investigations. The identified systems are analysed in terms of mechanical stability and elastic constants, $C_{ij}$, where the latter provide indication of in-plane vs. out-of-plane strength ($C_{11}$, $C_{33}$) and ductility ($C_{13}-C_{44}$, $C_{12}-C_{66}$). The superlattice ability to resist brittle cleavage along interfaces is estimated by Griffith’s formula for fracture toughness. The $\alpha /\alpha$-type TiB$_2$/MB$_2$ (M $=$ Mo, W), HfB$_2$/WB$_2$, VB$_2$/MB$_2$ (M $=$ Cr, Mo), NbB$_2$/MB$_2$ (M $=$ Mo, W), and $\alpha /\omega$-type AlB$_2$/MB$_2$ (M $=$ Nb, Ta, Mo, W), are suggested as the most promising candidates providing atomic-scale basis for enhanced toughness and resistance to crack growth.

## Introduction

Ab initio calculations pave the path for new design approaches allowing to suppress unwanted material’s behaviour in many applications, hence, are essential for accelerating modern technological processes. Especially in the field of thin ceramic films—including transition metal carbides, nitrides, and diborides—ab initio predictions are viewed as useful trend-givers^1–4^ and almost routinely complement experimental studies^5–8^. Our work focuses on transition metal diborides (MB$_2$s) which belong to ultra-high temperature ceramics (UHTC) and are attractive for high hardness, good resistance to abrasive and erosive wear as well as excellent oxidation and corrosion resistance^9–13^. On the atomic scale, these properties stem from strong covalent, ionic-covalent bonds between boron and transition metal atoms^1,14^ and, in case of thin films, can be ascribed also to the unique nanocomposite structure^5,6^. MB$_2$ thin films, however, show limited ability to plastically deform when subject to mechanical and thermal loads, which results in easy crack initiation/propagation and ultimately leads to permanent failure.

The last two decades brought several concepts to suppress brittle behaviour and crack propagation during deformation of thin ceramic films. The so-called “intrinsic” approaches applied to transition metal nitride, carbide, and diboride thin films are based on alloying on transition metal sublattice^15,16^, or vacancy-induced toughening^17,18^ which decrease the elastic stiffness (typically manifested by lower indentation modulus) and reduces the tendency to form cracks. Other “extrinsic” approaches are based on the formation of multilayered structures with spatial heterogeneity ensuring effective dissipation of the accumulated energy in the vicinity of a pre-existing crack. In superlattice structures, crack propagation is deflected and blunted by interfaces between flexible and stiff layers^19^, or the cracks are retarded by interfaces formed by a jump-like alternation of the growth direction during deposition (chevron-like morphology)^20^.

The concept of multilayers, especially in the form of periodically alternating coherent nanolayers called *superlattices* (SLs), is recognised as a powerful strategy for improving strength and toughness of thin film ceramics. Till today, attention has been paid primarily to the family of cubic transition metal nitride films where optimisation of the bilayer thickness (bilayer period, $\Lambda$) was shown to significantly improve mechanical properties^21–23^. According to widely accepted reasoning by^19^ and^24^, $\Lambda$ controls mobility of dislocations generated within individual SL layers. The dislocation glide across interfaces is hindered by coherency stresses (lattice mismatch, $\Delta a$) and the difference in the shear modulus of the individual layer materials ($\Delta G$). These two important strengthening effects produce a hardness increase, first reported for TiN/VN^25^ and TiN/NbN^24^ SLs with large lattice mismatch. When the bilayer period was set to 5 and 9 nm, respectively, these SLs exhibited a hardness increase of about 200% compared to their monolithic building blocks. Other nitride-based SL systems, TiN/WN^22^, TiN/V$_{0.6}$Nb$_{0.4}$N^26^, TiN/ZrAlN^27^, ZrN/Zr$_{0.63}$Al$_{0.37}$N^28^—where, in contrast, the lattice mismatch was essentially zero—were also reported to exhibit a hardness enhancement, ascribed to large differences in their shear moduli. Recent studies on TiN/CrN^23^, TiN/WN^22^, TiN/MoN^29^ SL films demonstrate that SL design via coherency strains, shear modulus mismatch of the layer components, as well as chemical and structural modulations at interfaces allows also for toughness enhancement. Performing micromechanical cantilever bending experiments, the authors report fracture toughness (K$_{IC}$) increase by tens of percent (at the *optimal* $\Lambda$).

Studies on nitride SLs raise expectations that SL-induced fracture toughness enhancement may be also achieved in transition metal diborides. The first proof-of-concept investigations on TiB$_2$/WB$_2$ and TiB$_2$/ZrB$_2$ magnetron sputtered SL films were conducted by Hahn et al. in 2023^30^. Here we employ high-throughput *ab initio* calculations to develop design guidelines for boride-based SLs with suitable atomic-scale basis for strength and ductility. The SL layer components include group 3–6 transition metals diborides, MB$_2$s, M $=$ Sc, Y (group 3); Ti, Zr, Hf (group 4); V, Nb, Ta (group 5); Cr, Mo, W (group 6), and a post-transition metal diboride, AlB$_2$ (group 13). Contrarily to transition metal nitrides with typical cubic (fcc) structure, borides crystallise in two types of hexagonal structure. Early MB$_2$s (M $=$ Y, Sc, Ti, Zr, Hf, V, Nb, Ta) show energetic preference for the AlB$_2$-type phase $\alpha$, space group $\#$191–P6/mmm), while late MB$_2$s (M $=$ Cr, Mo, W) crystallise in the W$_2$B$_{5-x}$-type phase ($\omega$, space group $\#$194–P6$_3$/mmc)^1^. The $\alpha$ and $\omega$ phase alternate metallic and boron layers stacked along the [0001] axis and differ only by the stacking of the metal planes and by the geometry of the boron hexagons which are either flat ($\alpha$) or puckered ($\omega$). We assume (semi-)coherent SL interfaces orthogonal to the [0001] axis. Following systematic screening of (1) $\alpha$ vs. $\omega$ phase preference of the constituent layer materials, (2) lattice and shear modulus mismatch, (3) ductility and strength indicators, as well as (4) theoretical fracture toughness values, $\alpha /\omega$-type TiB$_2$/MB$_2$, M $=$ (Mo, W), HfB$_2$/WB$_2$, VB$_2$/MB$_2$, M $=$ (Cr, Mo), NbB$_2$/MB$_2$, M $=$ (Mo, W), and $\alpha /\omega$-type AlB$_2$/MB$_2$, M $=$ (Nb, Ta, Mo, W), are proposed as the most promising SL candidates with optimal combination of mechanical properties.

## Methods

**Figure 1:**
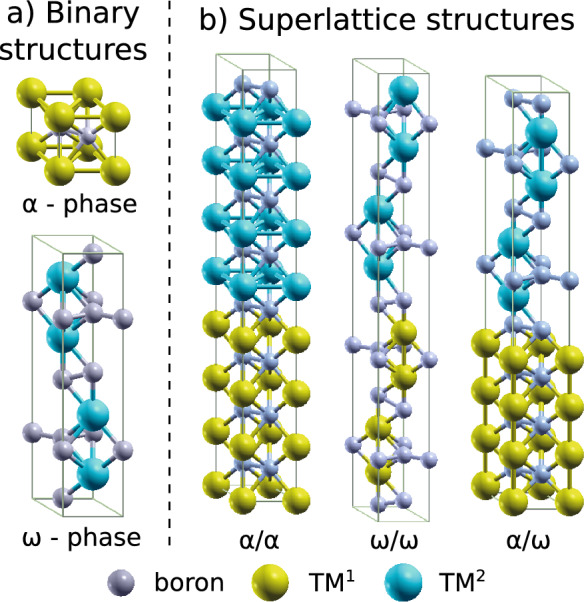
Illustration of supercells used to model (**a**) the $\alpha$ and $\omega$ phase of metal diborides, MB$_2$s, and (**b**) their $\alpha$/$\alpha$, $\omega$/$\omega$, $\alpha$/$\omega$ superlattice structures.

DFT calculations were carried out using QUANTUM ESPRESSO v. 6.4.1. employing the projector-augmented wave^31^ pseudopotentials and the Perdew-Burke-Ernzerhof parametrization of the electronic exchange-correlation functional^32,33^. Hexagonal binary structures of $\alpha$-MB$_2$ (P6/mmm) and $\omega$-MB$_2$ (P6$_3$/mmc) were modelled using the $\alpha$-AlB$_2$-type structure (3 atoms) and the $\omega$-W$_2$B$_{5-x}$-type structure (12 atoms), respectively. For SLs, combinations of 4 unit cells of $\alpha$ (1$\times$1$\times$4, 12 atoms) and 1 unit cell of $\omega$ (1$\times$1$\times$1, 12 atoms) were used as models for different $\alpha$/$\alpha$, $\omega$/$\omega$, $\alpha$/$\omega$ ($\omega$/$\alpha$) SL structures (Fig. 1). Total energies and structural parameters all MB$_2$s were evaluated by relaxing cell shapes, volumes and atomic coordinates. An 11$\times$11$\times$11, 11$\times$11$\times$9, and 11$\times$11$\times$5 *k*-point grid of the Brillouin zone was used for the $\alpha$-MB$_2$, $\omega$-MB$_2$, and SL systems, respectively, together with a plane wave energy cutoff of 90 Ry. The difference between total energies and forces for supercells at two consecutive self-consistent steps was always below 10$^{-7}$ Ry and 10$^{-5}$ Ry/bohr, respectively. All plane wave energy cutoffs and *k*-point meshes were carefully chosen to ensure a total energy convergence up to 5 meV/atom.

Chemical stability was quantified by the energy of formation^1^

1 $$\begin{aligned} E_{f} = \frac{1}{\sum _{i}}\left( E_{\text {tot}}-\sum _{i}n_i\mu _{i}\right) , \end{aligned}$$

where $E_{\text {tot}}$ is the total energy of the supercell, $n_i$ the number of atoms of type *i*, and $\mu _i$ are the chemical potentials of the individual elements in their ground state (at zero Kelvin), i.e. fcc-Al; bcc-V, -Nb, -Ta, -Cr, -Mo, -W; rhomboedral-B; and hcp-Ti, -Zr, -Hf. Furthermore, the interface energy of a SL

2 $$\begin{aligned} E_{\text {intf}}(SL)= \frac{1}{2A} \bigg ( E_{\text {tot}}(\text {SL}) - E_{\text {tot}}\left( \text {M}^{1}\text {B}_{2}\right) - E_{\text {tot}}\left( \text {M}^{2}\text {B}_{2}\right) \bigg ), \end{aligned}$$

was obtained by calculating the difference of the SL total energy, $E_{\text {tot}}(\text {SL})$, and total energy of the individual layer materials, $E_{\text {tot}}(\text {M}^{1}\text {B}_{2})$ and $E_{\text {tot}}(\text {M}^{2}\text {B}_{2})$, where the factor 2 in the denominator represents two created surfaces, *A*. Elastic constants (C$_{ij}$) were evaluated employing the THERMO-PW method^34^ in the QUANTUM ESPRESSO code and used to asses mechanical stability following Ref.^35^. From the obtained elastic constants, the polycrystalline bulk (*B*), shear (*G*), and Young’s modulus (*E*) were evaluated using the Voigt-Reuss-Hill approximation^36^.

## Results and discussion

### Identification of promising diboride combinations

To identify the most promising diboride-based SLs, we first focus on the individual layer materials—MB$_2$, M $=$ Sc, Y (group 3); Ti, Zr, Hf (group 4); V, Nb, Ta (group 5); Cr, Mo, W (group 6), and a post-transition metal, M $=$ Al (group 13)—and assess which out of their two competing phase prototypes ($\alpha$ vs. $\omega$) is/are energetically likely to form. Assuming (semi-)coherent SL interfaces orthogonal to the hexagonal [0001] axis, we further evaluate lattice and elastic mismatch between all MB$_2$ combinations and identify those providing a suitable basis for coherent interfaces and strength enhancement.

**Figure 2:**
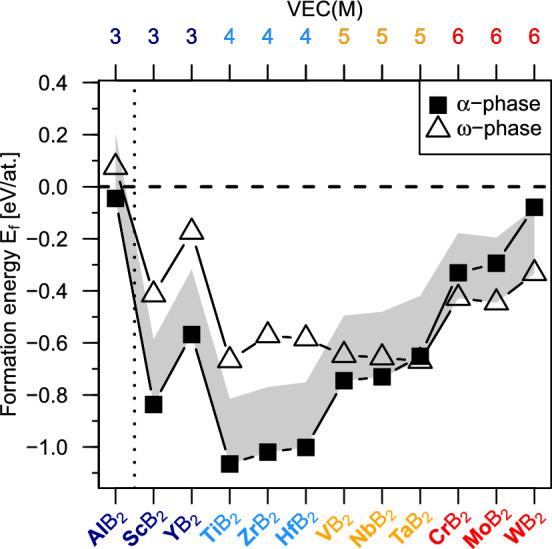
Formation energy, $E_f$, of metal diborides, MB$_2$, in the $\alpha$ and $\omega$ phase, grouped by the valence electron concentration of the metal atom, VEC(M). The grey-shaded region marks the threshold of our selection-criterion (0.25 eV/at.) of diboride-layer constituents. Phases outside the grey region are considered too unlikely to form and therefore discarded. Note that Al is not adjacent to the remaining M elements in the periodic table and has no d electrons.

For $n=12$ metals, there are $n(n-1)/2=66$ $\alpha /\alpha$ SLs (excluding SLs with the same transition metals, e.g. $\alpha$-AlB$_2$/$\alpha$-AlB$_2$). Similarly, there are 66 $\omega /\omega$ SLs and 132 $\alpha /\omega$ SLs (again, excluding combinations of the same transition metals, e.g. $\alpha$-AlB$_2$/$\omega$-AlB$_2$). In total, we consider $66+66+132=264$ SLs. First, we discard the energetically most unlikely diboride combinations based on their preference for the $\alpha$ vs. $\omega$ phase, as indicated by the energy of formation ($E_f$) in Fig. 2. We assume that if $E_f$ of the less stable phase prototype ($\alpha$ or $\omega$) is *too high*—i.e., above an arbitrarily chosen 0.25 eV/at. threshold (see the grey-shaded region in Fig. 2)—SLs containing layers of such phase are unlikely to form, thus not further considered. According to Fig. 2 and in agreement with literature^1^, group 3–4 transition metals (Sc, Y; Ti, Zr, Hf) show a strong energetic preference for $\alpha$-structured diborides, with $E_f\approx {(0.39}\text {--}0.45)$ eV/at. below that of the $\omega$ phase. Moreover, $\omega$-ScB$_2$ and $\omega$-YB$_2$ are mechanically unstable (based on the calculated elastic constants, following stability criteria by^35^). Changing to the group 5 transition metals (V, Nb, Ta), $E_f$ of the $\alpha$ phase increases and becomes close to that of the $\omega$ phase, with $E_f$ differences as small as 0.09 eV/at. Consequently, we consider both phase prototypes and argue that the metastable phase can form via the template effect induced by the second SL layer material, similar to e.g. interface-induced stabilisation of metastable cubic rocksalt-structure AlN in TiN/AlN SLs^21^. Importantly, TaB$_2$ is a turning point that exhibits almost overlapping $E_f$ values of the $\alpha$ and $\omega$ structure, $\omega$ being 0.01 eV/at. below $\alpha$. Following this trend, group 6 transition metals (Cr, Mo, W) preferentially form in the $\omega$ phase, and are overall less stable (less negative $E_f$ values) compared to their group 4–5 diboride counterparts. As the $\alpha$ variant of Cr-, Mo-, and WB$_2$ is still energetically close (with $E_f$ difference below 0.1 eV/at.), we again assume it may form in a SL due to the template effect. AlB$_2$ shows energetic preference for the $\alpha$ phase, with $E_f$ close to zero, while the energetically close $\omega$ allotrope yields already positive $E_f$ value indicating energetic instability. To sum up, based on Fig. 2 we assume that Al; Sc, Y; Ti, Zr, Hf can only form $\alpha$ diborides, while V, Nb, Ta; Cr, Mo, W can form both $\alpha$ and $\omega$ diborides, where the less preferred phase may be stabilised by SL interfaces. While this is out of the scope of the present work, we note that the $\alpha$ vs. $\omega$ phase preference may change due to the presence of boron or transition metal vacancies^1,7,37,38^, commonly formed in coatings prepared by physical vapour deposition (PVD) techniques.

**Figure 3:**
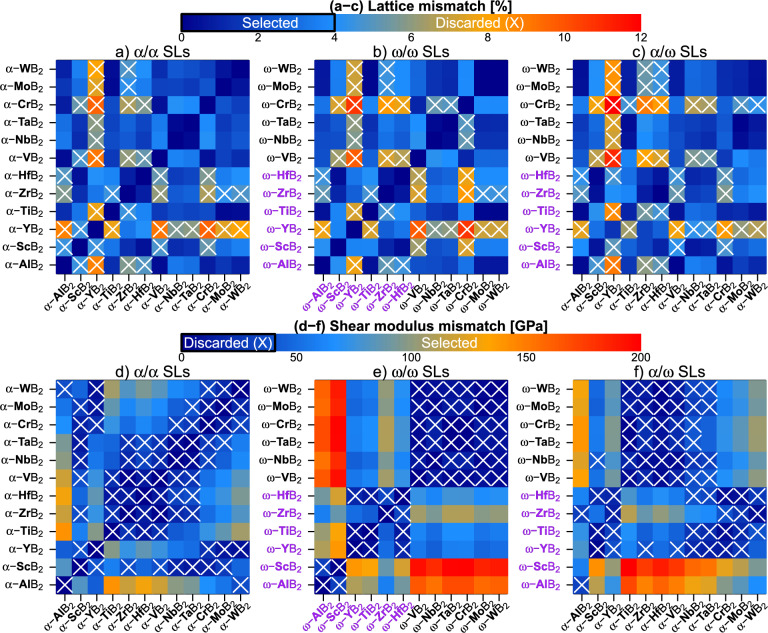
Lattice mismatch (**a**–**c**) and shear modulus mismatch (**d**–**f**) between diborides (in their $\alpha$ and $\omega$ phase), together with the applied selection limit, where the white crosses mark diboride combinations not satisfying the selection requirements. The purple-labelled diborides are energetically highly unlikely to form (c.f. Fig. 2). Note that panels a, b, d, and e always yield zero on the diagonal from the bottom left to top right (as e.g. $\alpha$-AlB$_2$ and $\alpha$-AlB$_2$ yield zero $\Delta a$) and are symmetric along this diagonal (as e.g. $\alpha$-AlB$_2$ and $\alpha$-TaB$_2$ yield the same $\Delta a$ as $\alpha$-TaB$_2$ and $\alpha$-AlB$_2$).

To pre-select promising material combinations for diboride SLs, Fig. 3 depicts lattice and shear modulus mismatch, $\Delta a$ and $\Delta G$, of 12 MB$_2$s in their $\alpha$ and $\omega$ phase variant. Based on $\Delta a$ and $\Delta G$, we identify SLs that (1) can be grown (semi-)coherently, as the in-plane lattice parameters of their constituent diborides do not largely differ ($\Delta a$), and (2) provide efficient obstacles to the dislocation motion by interface strains ($\Delta a$) or varying dislocation line energies ($\Delta G$)^19,24,26,39^. Inspired by $\Delta a$ and $\Delta G$ values for transition metal nitride SLs (see e.g. Refs.^22,23,29,40^), we set the following criteria

3 $$\begin{aligned} (\Delta a < 4\%)\,\, \& \,\, (\Delta G > 40\,\text {GPa}). \end{aligned}$$

According to Fig. 3a–c, a large lattice mismatch ($\Delta a>4\%$) is produced when combining YB$_2$ with any of the group 5–6 diborides, regardless of their phase modification. TiB$_2$ yields a plausible lattice mismatch when combined with almost any diboride with the exception of YB$_2$ and ZrB$_2$. In terms of our $\Delta a$ criterion, group 5–6 diborides (both $\alpha$ and $\omega$ structured) can be freely combined, with few exceptions including $\omega$-CrB$_2$. Regarding the shear modulus mismatch (Fig. 3d–f), our calculations reveal that combinations of $\alpha$-structured ZrB$_2$, HfB$_2$, VB$_2$, NbB$_2$, TaB$_2$ produce $\Delta G<40$ GPa, hence not provide a suitable basis for obstructing dislocation movement. The same is true for combinations of the group 6 $\alpha$-phased diborides between themselves. Furthermore, $\alpha$-TiB$_2$ exhibits $\Delta G>40$ GPa when combined with almost any $\alpha$ diboride with the exception of ZrB$_2$, HfB$_2$, and VB$_2$. Group 5–6 MB$_2$s in the $\omega$ structural variant exhibit essentially zero $\Delta G$ when combined with each other. Comparably low $\Delta G$ is predicted for combination so $\alpha$-structured TiB$_2$, ZrB$_2$, HfB$_2$, and VB$_2$ with $\omega$-structured MB$_2$ with M from from group 5–6.

Our lattice and shear modulus mismatch criteria (Eq. 3) yield 24 $\alpha /\alpha$, 28 $\omega /\omega$, and 47$\alpha /\omega$ SL candidates. Discarding SLs in which at least one layer forms in its highly unfavourable phase (e.g. $\omega$-AlB$_2$/$\omega$-TiB$_2$ or $\alpha$-ScB$_2$/$\omega$-ZrB$_2$, see $E_f$ results in Fig. 2) yields 24 $\alpha /\alpha$ and 22 $\alpha /\omega$ SLs. We note that possible candidates for $\omega /\omega$ SLs can only contain group 5–6 transition metals, as only these easily form the $\omega$ phase (c.f. Fig. 2). The group 5–6 MB$_2$s, however, are elastically very similar ($\Delta G\approx 0$ in Fig. 3e), which disqualifies $\omega /\omega$ SL candidates. The $\alpha /\omega$ combinations are further cut down to 9 SLs, as we consider combinations of $\alpha$-CrB$_2$, $\alpha$-MoB$_2$, or $\alpha$-WB$_2$ (all preferring the $\omega$ structure) with $\omega$ diborides from the group 5–6 (e.g. $\alpha$-CrB$_2$/$\omega$-NbB$_2$, $\alpha$-WB$_2$/$\omega$-TaB$_2$) energetically unlikely.

### Phenomenological indicators of superlattice ductility and strength

**Figure 4:**
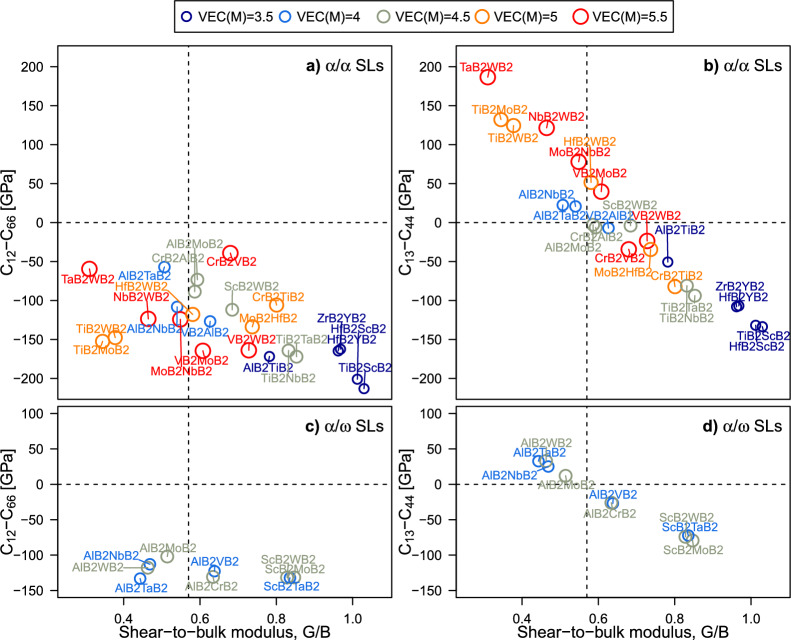
Ductility map for mechanically stable $\alpha$/$\alpha$ (**a**, **b**) and $\alpha$/$\omega$ (**c**, **d**) diboride SLs satisfying our stability, lattice and shear modulus mismatch criteria. The Cauchy pressure, $CP_{\parallel }=C_{12}-C_{66}$ (in-plane) and $CP_\perp =C_{13}-C_{44}$ (out-of-plane), is plotted against the shear-to-bulk modulus ratio (*G*/*B*). Dashed lines guide the eye for Pettifor’s^43^ and Pugh’s^44^ ductility criteria, while the symbol size and colour is based on the average valence electron concentration (VEC) of the M elements. Mind different *y*-axis ranges for (**a**, **b**) vs. (**c**, **d**).

**Figure 5:**
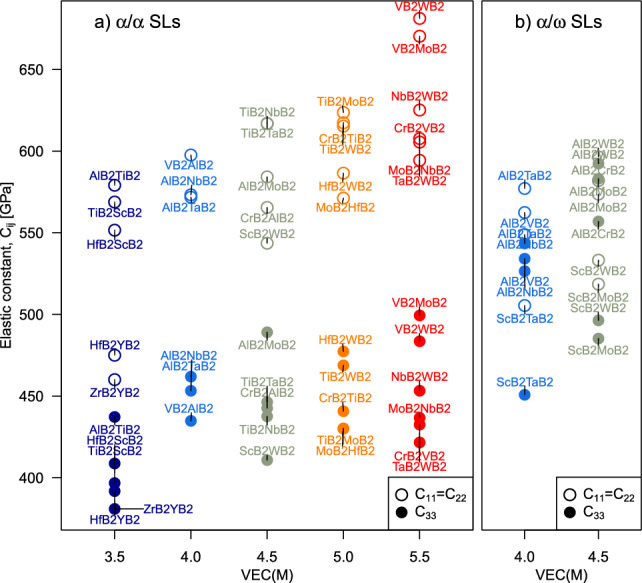
Strength indicators of (**a**) $\alpha /\alpha$ and (**b**) $\alpha /\omega$ SL candidates along the in-plane (open circles) vs. out-of-plane (filled circles) directions—estimated using the $C_{11}=C_{22}$ and $C_{33}$ elastic constants, respectively—and shown as a function of average valence electron concentration (VEC) of the metal sublattice.

Based on phase stability, lattice and elastic moduli mismatch criteria, of the initial 264 superlattice candidates, we consider 24 $\alpha /\alpha$ and 9 $\alpha /\omega$ combinations for more detailed investigations. Thus, we build the actual SL structures—assuming 1:1 ratio of the two diborides, with (semi-)coherent interfaces orthogonal to the [0001] axis, and bilayer thickness of about 4 times the *c* lattice parameter of the $\alpha$-AlB$_2$—and optimise their geometry by relaxing the supercell volume, shape, and ionic positions. The relaxation yields SL bilayer periods $\Lambda \approx {2.4}$–3.0 nm, where higher (smaller) values correspond to SLs containing Y (Cr, V) due to relatively large (small) *c* lattice parameter of YB$_2$ (CrB$_2$, VB$_2$). Having fully relaxed SL structures, their single-crystal elastic constants, $C_{ij}$, are calculated and used to assess (1) mechanical stability^35^ (not shown, all 24 $\alpha /\alpha$ and 9 $\alpha /\omega$ SLs are stable), as well as (2) ductility and strength phenomenological indicators (Figs. 4 and 5).

In Fig. 4, relative ductility of the SL candidates is assessed by comparing their shear-to-bulk modulus ratio (*G*/*B*) and Cauchy pressure (*CP*). Within the family of cubic transition metal nitrides^41,42^, $CP=C_{12}-C_{44}$ and *G*/*B* values have been often used as semi-empirical criteria to predict ductility. Proposed by Pettifor^43^ and Pugh^44^, these comparative criteria indicate that materials with larger positive *CP* and *G*/*B* values below 0.5 are likely to exhibit better ductility than materials with lower *CP* and larger *G*/*B*. More generally, high *CP* and low *G*/*B* can be understood as indicators of enhanced metallic character, which is an important contributing factor to plasticity at the atomic-scale. For transition metal diborides, their hexagonal symmetry dictates 5 independent elastic constants (since $C_{12}\ne C_{13}=C_{23}$; $C_{44}=C_{55}\ne C_{66}$), leading to two independent *CP* values^45^,

4 $$\begin{aligned} CP_{\parallel }&=C_{12}-C_{66}\,, \end{aligned}$$

5 $$\begin{aligned} CP_{\perp }&=C_{13}-C_{44}=C_{23}-C_{55}. \end{aligned}$$

These indicate the ability of the SL to respond in a more brittle or ductile manner when subject to in-plane ($\parallel$, i.e. within basal planes and parallel to interfaces) and out-of plane ($\perp$, i.e. within prismatic planes orthogonal to interfaces) shear deformation, respectively. As shown in Fig. 4a, c, the calculated in-plane $CP_{\parallel }$ Cauchy pressures suggest that the selected SLs have strongly directional bonding and are likely to exhibit brittle behaviour when subject to shear deformation within (0001) planes parallel to interfaces. The observation holds for both $\alpha /\alpha$ and $\alpha /\omega$ SL structures. In contrast, large positive $CP_{\perp }$ values in Fig. 4b, d suggest that part of the investigated superlattices possess good ductility when subject to shear deformation within prismatic planes. Ab initio calculations therefore point towards relatively low ability to accommodate shear stresses within the strongly-bonded honeycomb-structure boron layers (and hexagonal networks of transition metal layers) in contrast to relatively more ductile response to tilting of the SL [0001] axis, which induces (0001)$\langle \overline{1}2\overline{1}0\rangle$ and (0001)$\langle 10\overline{1}0\rangle$ shearing. The larger spread of $CP_{\perp }$ values also suggests that the SL response to shearing of the prismatic plane may be also more easily tuned by choosing different combinations of metal elements.

Excluding outliers as, e.g., AlB$_2$/TaB$_2$ or AlB$_2$/NbB$_2$, Fig. 4b uncovers a correlation between the average VEC of the metal sublattice and the Cauchy pressure $CP_{\perp }$ of the superlattice. This indicates an overall improvement in ductility as a function of the average VEC, which is consistent with trends reported by previous ab initio calculations of elastic constants of group 4–6 MB$_2$s^46^. In the supplemental material of that work, it has also been shown that the population of d states at the Fermi level increases with the material VEC. Thus, we infer that the reductions in shear resistance and (presumably) improved ductility of diboride SLs stems from enhanced occupation of d electronic states, analogous to what demonstrated for transition-metal nitrides under deformation^41,42,47^. Furthermore, some of the SLs that do not follow the $CP_{\perp }$ vs VEC trend in Fig. 4b contain AlB$_2$. The effect may be attributed to qualitatively different electronic structure of AlB$_2$ (Al does not have electrons in the d shell) compared to other MB$_2$s^48,49^. However, detailed chemical bonding analyses will be required to better elucidate the electronic mechanism underlying decreased shear resistance in transition metal diborides and their SLs.

Among the theoretically most ductile $\alpha$-structured SLs are TaB$_2$/WB$_2$, TiB$_2$/MoB$_2$, TiB$_2$/WB$_2$, NbB$_2$/WB$_2$, and MoB$_2$/NbB$_2$ SLs, with average VEC of the metal sublattice of 5 or 5.5. Thus, a good basis for ductility may be achieved by combining MB$_2$s from the group 5 and 6, e.g. TaB$_2$/WB$_2$, or a group 4 MB$_2$ with a group 6 MB$_2$, e.g. TiB$_2$/MoB$_2$. Changing to $\alpha /\omega$ SL candidates (Fig. 4c, d)—i.e. $\alpha$-AlB$_2$/$\omega$-MB$_2$, M$=$(V, Nb, Ta, Cr, Mo, W), and $\alpha$-ScB$_2$/$\omega$-MB$_2$, M$=$(Ta, Mo, W)—we see again that $CP_{\parallel }<0$ and $CP_{\parallel }<CP_{\perp }$. The most ductile SLs are $\alpha$-AlB$_2$/$\omega$-MB$_2$, M$=$(Nb, Ta, Mo, W), with average VEC(M) of 4 and 4.5. In contrast to $\alpha /\omega$ SL candidates, the $\alpha /\alpha$ ones show a larger spread of ductility indicators. Also, $\alpha /\alpha$ SLs that are predicted to be the most ductile exhibit significant anisotropy in Cauchy pressures, e.g. $CP_\parallel$ and $CP_\perp$ of the most ductile TaB$_2$/WB$_2$ SL is above 250 GPa, while it is $\approx {60}$ GPa for the most brittle TiB$_2$/ScB$_2$ SL.

In Fig. 5, we present $C_{11}$ and $C_{33}$ elastic constants of the same SLs as shown in Fig. 4 and visualise their dependence on average VEC of the metal sublattice. The $C_{11}$ and $C_{33}$ values are expected to correlate with the in-plane and out-of-plane (orthogonal to interfaces) tensile strength of the SL, respectively. This is because $C_{11}$ and $C_{33}$ equate to the slope of the stress vs. elongation relationship for small elastic strain. The larger the elastic constant value, the higher the tensile stress that the material withstands at a given strain. Similarly to ductility indicators (Fig. 4a, b) also strength indicators of $\alpha$-structured SLs (Fig. 5a, b) are significantly directional. Due to $C_{33}<C_{11}$, we predict lower strength orthogonal to SL interfaces, which is intuitive considering lower atom density and longer bonds along the [0001] direction. The difference between $C_{33}$ and $C_{11}$ is indeed pronounced—often above 100 GPa—as illustrated by e.g. AlB$_2$/ScB$_2$, TiB$_2$/NbB$_2$, or CrB$_2$/VB$_2$. It is also important to remark that both $C_{33}$ and $C_{11}$ values increase with average VEC of the metal sublattice. Among the elastically stiffest $\alpha$-based SLs are VB$_2$/MoB$_2$, VB$_2$/WB$_2$, NbB$_2$/WB$_2$, TiB$_2$/CrB$_2$, TiB$_2$/MoB$_2$, TiB$_2$/WB$_2$, where TiB$_2$/MoB$_2$, TiB$_2$/WB$_2$, and NbB$_2$/WB$_2$ were also identified as ductile materials (Fig. 4b). Strength indicators calculated for $\alpha /\omega$ SLs (Fig. 5b) show less pronounced directional dependence, as the $C_{11}$ and $C_{33}$ elastic constants generally differ less. Although $C_{33}$ is typically lower than $C_{11}$, there are cases of $C_{33}$ almost equal to $C_{11}$, such as for $\alpha$-AlB$_2$/$\omega$-WB$_2$ or $\alpha$-AlB$_2$/$\omega$-MoB$_2$. Similar to $\alpha$-structured SLs, the $\alpha /\omega$ SLs show increasing elastic stiffness with increasing VEC, with the potentially hardest ones being $\alpha$-AlB$_2$/$\omega$-CrB$_2$, $\alpha$-AlB$_2$/$\omega$-MoB$_2$, and $\alpha$-AlB$_2$/$\omega$-WB$_2$. The latter two were also predicted to posses a suitable basis for ductility (Fig. 4d).

### Superlattice theoretical fracture toughness

Having discussed ductility (Fig. 4) and strength indicators (Fig. 5), we additionally assess the SL ability to resist brittle fracture along interfaces. Considering that the SLs exhibit lower out-of-plane than in-plane (parallel to interfaces) strength—indicated by relatively low $C_{33}$ elastic constants in Fig. 5a—we hypothesise that interfaces are among the easiest cleavage planes. This would be similar to what has been observed for transition metal nitride based AlN/TiN^50,51^ and AlN/VN SLs^51^. The Griffith’s formula for fracture toughness, $K_{1C}$^52^,

6 $$\begin{aligned} K_{1C}(0001) = \sqrt{4\cdot E^{\text {sep}}_{(0001)}E_{[0001]}}, \end{aligned}$$

is employed, where $E^{\text {sep}}_{(0001)}$ is the separation energy of the boron and metal planes (at the interface) and $E_{[0001]}$ the Young’s modulus in the [0001] direction. Note, however, that Eq. (6) provides an estimate of the fracture toughness. The formula is based on linear elastic fracture mechanics considerations and neglects possible plastic deformation mechanisms. Furthermore, as experimentally demonstrated for some transition metal nitride SLs (e.g. TiN/CrN^23^, TiN/WN^22^, TiN/MoN^29^), $K_{1C}$ may show a peak for bilayer periods typically around 5–15 nm, which are larger than the bilayer periods of our SL models. Besides, $K_{1C}$ values attainable in lab-scale physical vapour deposited (PVD) SL films also include effects of native point and line defects, as well as of grain boundaries for the case of polycrystalline samples.

**Figure 6:**
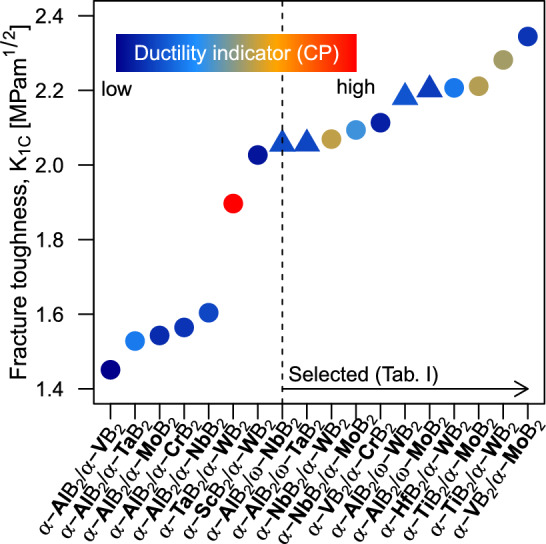
Theoretical fracture toughness ($K_{1C}$) of the most promising $\alpha /\alpha$ (circles) and $\alpha /\omega$ (triangles) diboride SLs—meeting energetic, lattice mismatch, and elastic mismatch criteria from section “Introduction”—visualised together with their ductility indicator based on the effective Cauchy pressure (CP). The most brittle SL (with CP$<-50$ GPa) are excluded. The dashed vertical lines marks selected SLs for which we present additional properties in Table 1.

Figure 6 presents the calculated $K_{1C}(0001)$ for the most promising SLs satisfying phase stability, lattice and shear modulus mismatch criteria discussed in previous Sections. From Griffith’s formula (Eq. (6)), fracture toughness is proportional to the directional Young’s modulus, hence directly incorporates the effect of our $C_{33}$ strength indicator. The expression, however, does not account for plastic deformation mechanisms, which are inherent to the ductile behaviour of the material. Plastic deformation would, in general, increase the actual fracture toughness by dissipating stress at a crack front. In Fig. 6 we forecast SL ductility using an *effective* Cauchy pressure, i.e., computed as $(CP_\parallel +CP_\perp CP_\perp )/3$. The SLs with effective *CP* below $-50$ GPa are omitted due to expected high brittleness. The results in Fig. 6 suggest that $\alpha$-structured AlB$_2$/MB$_2$ SLs, M$=$(V, Nb, Ta, Cr, Mo), possess the lowest fracture toughness and rather low ductility. Interestingly, the $K_{1C}(0001)$ of AlB$_2$/MoB$_2$ and AlB$_2$/WB$_2$ SLs significantly increases when the MoB$_2$ and WB$_2$ layers are considered in their (energetically more stable) $\omega$ phase variant. SLs exhibiting the highest $K_{1C}(0001)$ include $\alpha$-structured VB$_2$/MoB$_2$, TiB$_2$/WB$_2$, TiB$_2$/MoB$_2$, HfB$_2$/WB$_2$, where especially TiB$_2$/WB$_2$, TiB$_2$/MoB$_2$ provide a good basis for ductility.

### Suggestions of the most promising systems

Based on ab initio predictions of $K_{1C}(0001)$ (Fig. 6), we propose 11 most promising diboride SL candidates with optimal combination of fracture resistance and plasticity. Energetic, structural, and mechanical properties of these 11 systems are summarised in Table 1. We remind the reader that the selection is also based on energy stability, mechanical stability, lattice parameter and shear moduli mismatch between constituent layers.

In terms of formation energies, the SLs $\alpha$-AlB$_2$/$\omega$-MB$_2$, M $=$ (Nb, Ta, Mo, W), are the least stable, which can be attributed to high $E_f$ of $\alpha$-AlB$_2$ (Fig. 2). While exhibiting rather low lattice mismatch (0.4–1.8%), the shear modulus mismatch always exceeds 100 GPa, hence, should greatly contribute to hindering dislocation motion across interfaces and enhance hardness. However, $\alpha$-AlB$_2$/$\omega$-MB$_2$, M $=$ (Nb, Ta, Mo, W) SLs can be considered as the least ductile among the proposed 11 candidates due to negative $CP_\parallel$ and rather low $CP_\perp$ Cauchy pressures. The energetically more stable $\alpha$/$\alpha$ SLs yield lattice mismatch ranging from 0.2% (TiB$_2$/MoB$_2$) to 3.9% (NbB$_2$/MoB$_2$) and shear modulus mismatch ranging from 31 GPa (NbB$_2$/MoB$_2$) to 101 GPa (TiB$_2$/WB$_2$). Exception made for VB$_2$/CrB$_2$—for which we obtain $CP_\parallel \approx CP_\perp$—the $\alpha$/$\alpha$ SLs are more anisotropic than the $\alpha$/$\omega$ SLs, as they display larger differences between $CP_\parallel$ and $CP_\perp$ values. Their $CP_\perp$ can exceed 100 GPa, as shown by results collected for TiB$_2$/MoB$_2$, TiB$_2$/WB$_2$, and NbB$_2$/WB$_2$. Thus, $\alpha$/$\alpha$ TiB$_2$/MoB$_2$, TiB$_2$/WB$_2$, and NbB$_2$/WB$_2$ SLs are expected to exhibit improved ductility in comparison to the other proposed candidates. The polycrystalline Young’s moduli of $\alpha$/$\alpha$ SLs also display a relatively large spread compared to those calculated for $\alpha$/$\omega$ candidates. The values range from 260–280 GPa for TiB$_2$/MoB$_2$ and TiB$_2$/WB$_2$ up to 475 GPa calculated for the rather brittle and most elastically isotropic VB$_2$/CrB$_2$.

Table 1 also indicates whether the strength and/or ductility of the proposed SLs exceeds that of constituent layer materials. This is assessed by comparing $C_{11}$, $C_{33}$ elastic constants (strength indicators) and $CP_{\perp }$, $CP_{\parallel }$ values (ductility indicators) to that of the binary constituents. All SL candidates in Table 1, except $\alpha$-$\alpha$ TiB$_2$/WB$_2$, possess higher $C_{11}$ and $C_{33}$ elastic constants compared to their binary building blocks. Also in terms of ductility indicators, the $CP_{\perp }$ of the SLs exceeds that of the layer constituents. The $CP_{\parallel }$ values are quite similar. We note that the values of elastic constants may vary as a function of the bilayer period, thus possibly affecting trends in mechanical properties described in previous Sections. Our predictions are based on bilayer periods of $\approx {2.4}\text {--}2.8$ nm.

In addition to information concerning mechanical properties, Table 1 lists interface energies ($E_{\text {intf}}$ calculated using Eq. (2)), which are estimates of the energetic cost for interface formation. A small/high positive $E_{\text {intf}}$ value indicates low/high energy required to form interfaces, whereas a negative $E_{\text {intf}}$ suggests energetic gain upon interface formation. Highly negative $E_{\text {intf}}$ may be ascribed to substantial interface-induced structural relaxations and thus indicate that the diboride constituent phases are not valid reference states for evaluation of the interface energy. Most $\alpha /\alpha$ SL candidates yield small and slightly negative $E_{\text {intf}}$ ranging from $-\,0.181$ to $-\,0.032$ eV/atom. At variance with other $\alpha /\alpha$ structure SLs, the systems HfB$_2$/WB$_2$ and VB$_2$/MoB$_2$ exhibit rather large positive ($E_{\text {intf}}\approx {0.247}$ eV/at.) and negative ($E_{\text {intf}}\approx {-\,0.243}$ eV/at.) interface energies. Furthermore, $\alpha /\omega$ SLs exhibit very high $E_{\text {intf}}$ of $\approx {0.462}$–0.668  eV/at., which may stem from poor energetic stability of $\alpha$-AlB$_2$ (Fig. 2). In view of these results, it is plausible to expect that the proposed $\alpha /\omega$ SL systems are considerably more difficult to be synthesised than $\alpha /\alpha$ SL structures.

To summarise, among the top SL candidates, $\alpha$-structured TiB$_2$/MB$_2$ SLs, M$=$(Mo, W), offer the highest energetic stability in combination with high shear modulus mismatch (83–101 GPa), which may result in superior hardness. The TiB$_2$-containing systems have negligible lattice disregistry ($\Delta a\approx {0}$), show small interface energies, and are highly elastically anisotropic: expected to display high in-plane stiffness combined with good out-of-plane ductility. Contrarily, the HfB$_2$/WB$_2$ SL provides coherency stresses via 3.8% lattice mismatch and exhibits lower anisotropy (still being considerably stiffer in-plane). Finally, the $\alpha$-phase VB$_2$/CrB$_2$ SL is predicted to be the most elastically isotropic system. It has very small lattice mismatch together with low interface energy and $\Delta G\approx {50}$ GPa.

**Table 1:** Selected properties of the proposed most promising SL candidates: energy of formation, $E_f$ (in eV/at.); lattice and shear modulus mismatch, $\Delta a$ and $\Delta G$ of the layer components (in % and GPa, respectively); polycrystalline bulk, shear, and Young’s modulus, *B*, *G*, and *E* (in GPa); Cauchy pressure, $CP_{\parallel }$ and $CP_{\perp }$ (in GPa); fracture toughness, $K_{1C}$ (0001) (in MPa$\sqrt{\text {m}}$); comparison between strength and ductility indicators, $\Delta C_{11}$, $\Delta C_{33}$ elastic constant (in GPa) and directional Cauchy pressure, $\Delta CP_{\parallel }$ and $\Delta CP_{\perp }$ (in GPa) of the superlattice and its diboride components, where $\checkmark$ ($\times$) denotes increase (decrease) in value, therefore increased (decreased) strength and ductility compared to diboride components; and interface energy (in J/m$^{2}$).

SL	Type	$E_f$	$\Delta a$	$\Delta G$	*B*	*G*	*E*	$CP_{\parallel }$	$CP_{\perp }$	$K_{1C}$	$\Delta C_{11}$	$\Delta C_{33}$	$\Delta CP_{\parallel }$	$\Delta CP_{\perp }$	$E_{\text {intf}}$
TiB$_{2}$/MoB$_2$	$\alpha /\alpha$	$-\,0.679$	0.22	83	276	95	256	$-\,152$	132	2.21	$\checkmark$	$\checkmark$	$\times$	$\checkmark$	$-\,0.028$
TiB$_{2}$/WB$_2$	$\alpha /\alpha$	$-\,0.579$	0.32	101	280	106	282	$-\,148$	125	2.28	$\times$	$\checkmark$	$\times$	$\checkmark$	$-\,0.181$
HfB$_{2}$/WB$_2$	$\alpha /\alpha$	$-\,0.526$	3.81	91	282	164	412	$-\,118$	52	2.21	$\checkmark$	$\checkmark$	$\checkmark$	$\checkmark$	0.247
VB$_{2}$/CrB$_2$	$\alpha /\alpha$	$-\,0.538$	0.46	50	286	194	475	$-\,39$	$-\,34$	2.11	$\checkmark$	$\checkmark$	$\checkmark$	$\checkmark$	$-0.042$
VB$_{2}$/MoB$_2$	$\alpha /\alpha$	$-\,0.529$	0.46	50	294	179	445	$-\,165$	40	2.34	$\checkmark$	$\checkmark$	$\times$	$\checkmark$	$-\,0.243$
NbB$_{2}$/MoB$_2$	$\alpha /\alpha$	$-\,0.511$	3.87	31	295	162	410	$-\,124$	78	2.09	$\checkmark$	$\checkmark$	$\times$	$\checkmark$	$-\,0.032$
NbB$_{2}$/WB$_2$	$\alpha /\alpha$	$-\,0.404$	1.74	43	305	142	368	$-\,124$	122	2.07	$\checkmark$	$\checkmark$	$\times$	$\checkmark$	$-0.053$
AlB$_{2}$/NbB$_2$	$\alpha /\omega$	$-\,0.324$	1.75	119	229	107	278	$-\,113$	25	2.06	$\checkmark$	$\checkmark$	$\times$	$\checkmark$	0.514
AlB$_{2}$/TaB$_2$	$\alpha /\omega$	$-\,0.325$	1.51	134	237	105	274	$-\,133$	33	2.06	$\checkmark$	$\checkmark$	$\times$	$\checkmark$	0.668
AlB$_{2}$/MoB$_2$	$\alpha /\omega$	$-\,0.166$	0.44	122	245	126	323	$-\,102$	12	2.18	$\checkmark$	$\checkmark$	$\checkmark$	$\checkmark$	0.360
AlB$_{2}$/WB$_2$	$\alpha /\omega$	$-\,0.227$	0.41	126	255	118	307	$-\,118$	33	2.20	$\checkmark$	$\checkmark$	$\times$	$\checkmark$	0.462

## Summary and conclusions

High-throughput first-principles screening was performed to pre-select candidates for diboride-based superlattices with optimal combination of fracture resistance and plasticity. Our calculations point towards the $\alpha$-structured TiB$_2$/MB$_2$ (M $=$ Mo, W) and HfB$_2$/WB$_2$ as to the most promising systems to start with.

First, considering all $\alpha$- and $\omega$-phased diborides of the group 3–6 transition metals together with AlB$_2$ as layer constituents resulted in 264 possible SL systems with $\alpha$/$\alpha$, $\alpha$/$\omega$, and $\omega$/$\omega$ layers. Selection based on energetic preference of the diboride layer for the $\alpha$ vs. $\omega$ phase, formation energies of $\alpha$/$\alpha$- and $\alpha$/$\omega$-structured SLs, lattice and shear modulus mismatch narrowed the focus to 33 SL candidates. These were modelled with sharp (0001) interfaces and bilayer periods of 2.4–3.0 nm (after relaxation). Phenomenological indicators of tensile strength and ductility (here the $C_{11}$ and $C_{33}$ elastic constants and directional Cauchy pressures) inferred that all SLs possess extremely high stiffness with respect to deformation of honeycomb-structure boron layers. Furthermore, some $\alpha$/$\alpha$-structured SLs showed ductility with respect to shearing of prismatic planes (orthogonal to interfaces), indicating relatively facile slip on (0001)$\langle \overline{1}2\overline{1}0\rangle$ and (0001)$\langle 10\overline{1}0\rangle$ systems. Finally, the SL resistance to brittle fracture along interfaces—estimated by fracture toughness $K_{1C}(0001)$—rendered 11 perspective SL candidates: $\alpha /\alpha$-type TiB$_2$/MB$_2$ (M$=$Mo, W), HfB$_2$/WB$_2$, VB$_2$/MB$_2$ (M$=$Cr, Mo), NbB$_2$/MB$_2$ (M$=$Mo, W), and $\alpha /\omega$-type AlB$_2$/MB$_2$ (M$=$Nb, Ta, Mo, W), further compared in terms of formation and interface energy, elastic moduli, elastic anisotropy, and strengthening/toughening effects of the interface. The proposed most promising TiB$_2$/MB$_2$ SLs (M$=$Mo, W) posses a negligible lattice mismatch and are highly elastically anisotropic. Other interesting SL candidate, HfB$_2$/WB$_2$, offers higher elastic isotropy.

In conclusions, our systematic high-throughput screening set the stage for in-depth investigations on specific SL systems. Though possibly metastable, the here-predicted SLs are likely accessible by e.g. non-equilibrium vapour physical deposition (PVD) techniques. Future research should address finite temperature effects on phase stability and mechanical properties. Expectedly, elevated temperatures may bring more competing phases into game, promote vacancy formation and boron diffusion across SL interfaces. To efficiently filter out the least promising SLs in our study, atomic-scale strength and ductility indicators used quantities derived only from zero Kelvin single-crystal elastic constants. Follow-up simulations should go beyond length and timescales of first-principles methods (e.g. classical molecular dynamics) and consider extended defects, having implications for plasticity and fracture mechanisms of materials and the macroscale.

## Data Availability

The data supporting the findings of this study are available from corresponding author upon reasonable request.
